# Supplementary material for: Extending Single Cell Bioprinting from Femtosecond to Picosecond Laser Pulse Durations
Source: Micromachines (Basel). 2021 Sep 29;12(10):1172. doi: 10.3390/mi12101172 (PMC8538086; doi:10.3390/mi12101172)
Supplement: Supplementary file 1 [file micromachines-12-01172-s001.zip › micromachines-1355383-supplementary.pdf]

micromachines

Article

Single-cell bioprinting by using femto- and picosecond laser pulses

Supplementary Materials:

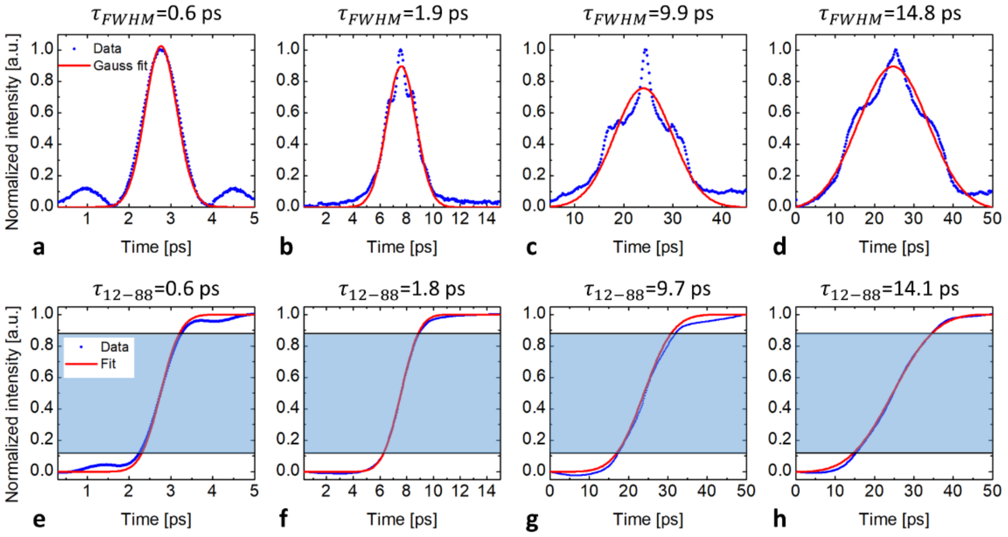

Figure S1. (a-d) Temporal pulse shapes (blue dots) were measured by an autocorrelator (PulseCheck, APE). The red lines represent Gauss fits to the data, facilitating the determination of full width at half maximum (FWHM) values. (e-h) However, for unsymmetrical or non-Gaussian intensity distributions, the FWHM pulse duration may alternatively be determined by the integrated curves as temporal distance between the 0.12 and the 0.88 levels. The fact that both methods yield pulse duration values which are equal within 5% deviation highlights that the pulse form is in fairly good agreement with a Gaussian distribution.

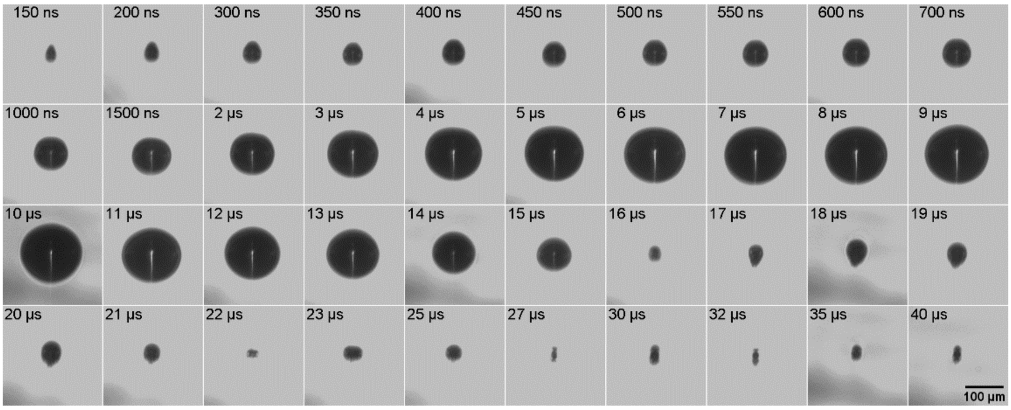

Figure S2. Time-resolved images of the cavitation bubble by using 1.8 ps pulse duration.

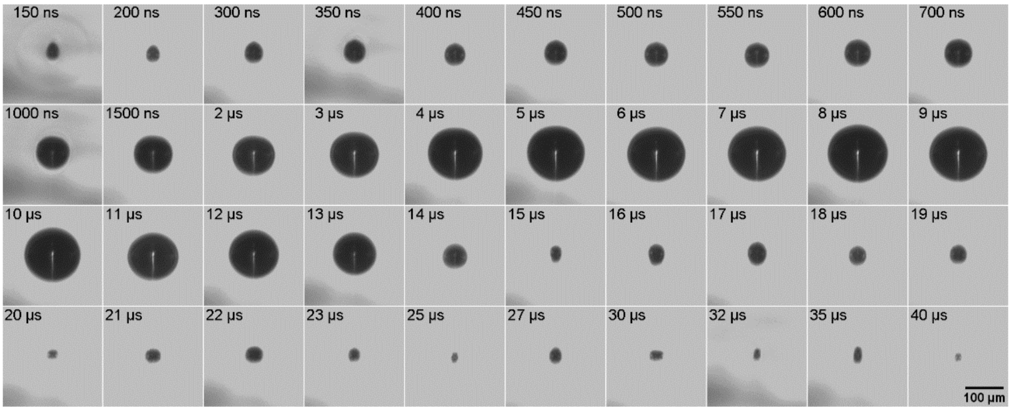

Figure S3. Time-resolved images of the cavitation bubble by using a 9.7 ps pulse duration.

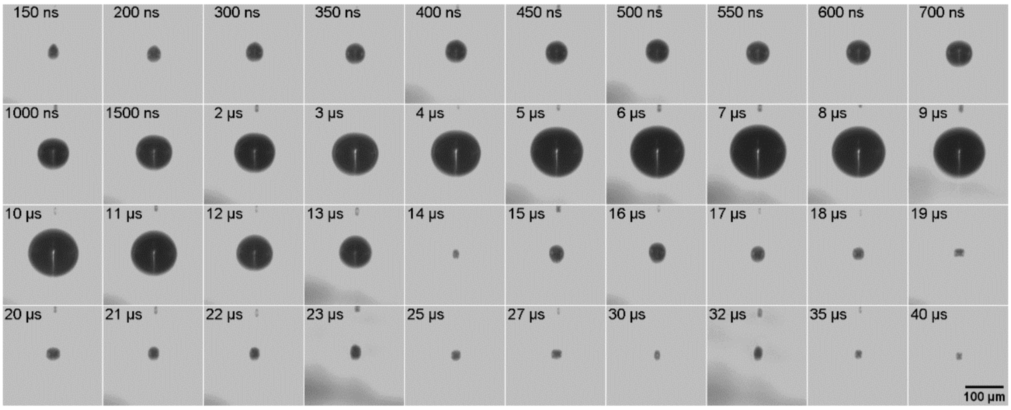

Figure S4. Time-resolved images of the cavitation bubble by using a 14.1 ps pulse duration.

Table S1. **Cell survival 15 min after transfer of hMSC cells at different pulse duration, laser pulse energies and focus depths. 275 out of 281 transferred cells survived the transfer in 16 independent experiments.**

	**Laser focus depth [μm] and laser pulse energy [μJ]**			
**τ [ps]**	**39 μm 1.5 μJ**	**52 μm 2 μJ**	**52 μm 2.5 μJ**	**65 μm 3 μJ**
0.6	16/18	18/18	16/16	19/19
1.8	18/19	20/21	8/8	19/19
9.7	17/18	17/17	16/16	21/21
14.1	19/19	17/17	18/18	16/17

Table S2. **Optical breakdown threshold energy E_{thr} depends on laser pulse duration and the ratio of pulse energy and breakdown threshold energy $\beta = E/E_{thr}$. The incident laser pulse energy was fixed at 2 μJ.**

**τ [ps]**	**E_{thr} [nJ]**	**β**
0.6	122	16.4
1.8	138	14.5
9.7	253	7.9
14.1	277	7.2
